# A Whey Fraction Rich in Immunoglobulin G Combined with *Bifidobacterium longum* subsp. *infantis* ATCC 15697 Exhibits Synergistic Effects against *Campylobacter jejuni*

**DOI:** 10.3390/ijms21134632

**Published:** 2020-06-29

**Authors:** Erinn M. Quinn, Michelle Kilcoyne, Dan Walsh, Lokesh Joshi, Rita M. Hickey

**Affiliations:** 1 Teagasc Food Research Centre, Moorepark, Fermoy, Co., P61 C996 Cork, Ireland; 2 Advanced Glycoscience Research Cluster, National Centre for Biomedical Engineering Science, National University of Ireland Galway, H91 TK33 Galway, Ireland; Michelle.Kilcoyne@nuigalway.ie (M.K.); lokesh.joshi@nuigalway.ie (L.J.); 3 Department of Microbiology, University College Cork, Co., T12YT20 Cork, Ireland; DWalsh@ucc.ie

**Keywords:** *Bifidobacterium*, *Campylobacteria*, adhesion, immunoglobulins, synbiotics, HT-29 cells

## Abstract

Evidence that whey proteins and peptides have health benefits beyond basic infant nutrition has increased dramatically in recent years. Previously, we demonstrated that a whey-derived immunoglobulin G-enriched powder (IGEP) enhanced adhesion of *Bifidobacterium longum* subsp. *infantis* ATCC 15697 (*B. infantis*) to HT-29 cells. In this study, we investigated the synergistic effect of IGEP-treated *B. infantis* on preventing the attachment of highly invasive *Campylobacter jejuni* 81–176 (*C. jejuni*) to intestinal HT-29 cells. The combination decreased the adherence of *C. jejuni* to the HT-29 cells by an average of 48% compared to the control (non-IGEP-treated *B. infantis*). We also confirmed that treatment of IGEP with sodium metaperiodate, which disables the biological recognition of the conjugated oligosaccharides, reduced adhesion of *B. infantis* to the intestinal cells. Thus, glycosylation of the IGEP components may be important in enhancing *B. infantis* adhesion. Interestingly, an increased adhesion phenotype was not observed when *B. infantis* was treated with bovine serum-derived IgG, suggesting that bioactivity was unique to milk-derived immunoglobulin-rich powders. Notably, IGEP did not induce growth of *B. infantis* within a 24 hours incubation period, as demonstrated by growth curves and metabolite analysis. The current study provides insight into the functionality of bovine whey components and highlights their potential in positively impacting the development of a healthy microbiota.

## 1. Introduction

Whey derived from cows’ milk contains many similar components to those found in human milk and for this reason, is a key ingredient in a wide variety of infant formulas. While breastfeeding is preferred, infant formulas containing whey proteins are currently the best alternative when breastfeeding is not an option [1]. Emerging evidence from in vitro, animal, and a small number of human studies indicate that a variety of beneficial bioactivities are linked to whey protein and its derivative forms, i.e., concentrate, isolate, hydrolysate, and individual proteins and peptides [1]. Whey proteins are suggested to play a role in influencing the infant gut microbiota. Bifidobacteria are strong colonizers of the infant gut and an array of bifidobacterial strains can utilize milk glycans as substrates for growth [2,3,4,5]. High levels of beneficial bacterial species such as bifidobacteria are present in the breast-fed infants’ gut, inhibiting the growth of pathogenic microorganisms, modulating the mucosal barrier function, and promoting inflammatory and immunological responses [6]. Balmer et al. [7] showed that infants ingesting a whey-protein formula had higher levels of bifidobacteria in their stool compared to those who received a casein-predominant formula at two weeks of age [7]. Similarly, a German-based double-blind study randomized 102 healthy infants under two weeks of age and demonstrated that whey protein-fed infants had more abundant counts of bifidobacteria in their stools [8]. More recently, it has been shown that infants receiving a formula low in protein and phosphate with whey protein as the main constituent developed a bifidobacteria profile similar to that of the breast-fed infants [9]. These studies indicate that supplementation of infant formula with specific whey proteins may have the potential to emulate the bioactivities associated with human breast milk, including the development of microbiota rich in beneficial bifidobacteria.

Breast milk and its constituents naturally select for protective bifidobacteria, and specific glycans present in mammalian milk have been demonstrated to bind to and inhibit a range of enteric pathogens in vitro [10,11,12]. Specific glycosylated milk components have been suggested to not only confer prebiotic effects, but also contribute to the colonization potential of bifidobacteria spp. in the intestine by directly modulating the intestinal cells, or by enhancing the adherence properties of the bacteria. For instance, our group recently demonstrated a bovine milk-derived fraction containing 23.64 μg/mL IgG among other components, including 3′- and 6′-sialyllactose, β-lactoglobulin, and α-lactalbumin, and was capable of modulating HT-29 cells which resulted in a subsequent increase in bifibacterial adhesion of up to 52-fold compared to the non-treated HT-29 cells [13]. Transcriptomic, proteomic, and glycomic analysis of the intestinal cells following treatment with the bovine milk-derived fraction confirmed that the cell surface proteome and glycome were altered [13,14]. Regarding enhancing the adherence abilities of bifidobacteria, growth of *B. longum* in defatted human milk upregulated a gene encoding a putative type II glycoprotein-binding fimbriae, which may be involved in attachment and colonization [15]. Chichlowski et al. [16] demonstrated the increased adherence of *B. longum* subsp. *infantis* ATCC 15697 (*B. infantis* to HT-29 intestinal cells following its growth on human milk oligosaccharides. Additionally, our group demonstrated that treatment of *B. infantis* with a combination of the milk oligosaccharides, 3′-and 6′-sialyllactose significantly increased bacterial adhesion to HT-29 cells up to 9.8-fold [17]. Previously, we also demonstrated the increased adhesion of *B. infantis* following exposure to a panel of oligosaccharides and a bovine whey-derived powder enriched in IgG [18]. Subsequent studies with goat milk oligosaccharides (GMO) indicated a prophylactic protective effect against *Campylobacter jejuni* colonization when HT-29 cells were pre-exposed to GMO-treated bifidobacteria [19].

Taken together, these studies indicate that glycosylated fractions from domestic animal milks may result in an increase in bifidobacteria colonizing the gut. The term synbiotic is used to describe the use of a combination of probiotics and prebiotics that together beneficially improve host health by modulating the development of a healthy gut microbiota. Therefore, the current study aimed to examine the effect of combining *B. infantis* with a bovine whey-derived fraction rich in IgG on the attachment of *B. infantis* to intestinal cells and in turn the ability of the combination to exclude an invasive strain of *C. jejuni* to intestinal cells in vitro.

## 2. Results

### 2.1. Characterization of Immunoglobulin G-enriched Powder (IGEP)

**Figure 1:**
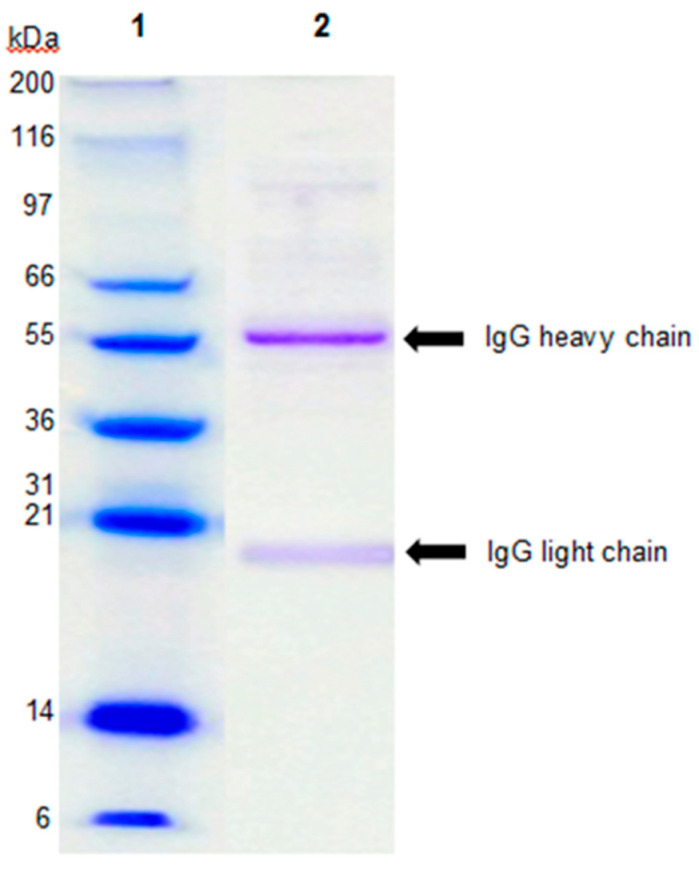
Protein characterization of the IgG-enriched powder (IGEP) by NuPAGE 4–12% SDS-PAGE separation with Coomassie Blue staining. Lane 1, molecular mass marker; Lane 2, reduced IGEP.

IGEP was kindly provided by Upfront technologies. The IGEP contained 77% protein as determined by the Bradford assay [20]. SDS-PAGE analysis of the IGEP powder (Figure 1) revealed that it mainly consisted of IgG, with two protein bands corresponding to 55 and 20 kDa representing the IgG heavy chain and light chain, respectively.

### 2.2. Effect of IGEP on B. infantis Adhesion

**Figure 2:**
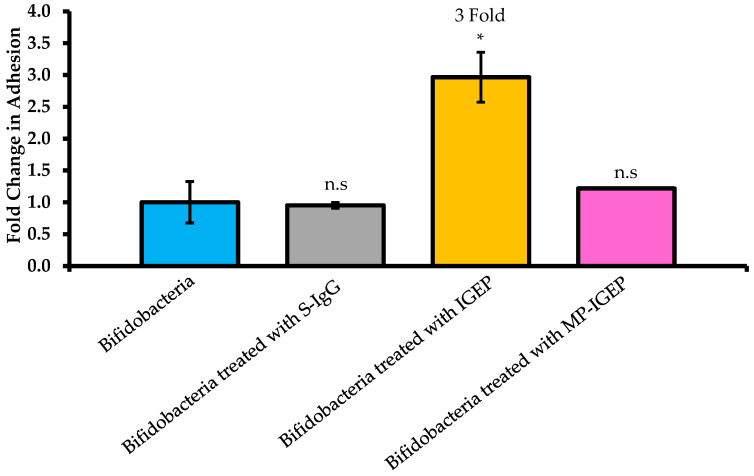
Adhesion of *Bifidobacterium infantis* to HT-29 cells following incubation with bovine serum-derived IgG (S-IgG), whey-derived Ig enriched powder (IGEP), and metaperiodate-treated IGEP (MP-IGEP). Results are from three biological replicate experiments, each performed in technical triplicate, except for S-IgG and MP-IGEP treatment which were assayed in technical triplicate using one biological replicate. Results were calculated as the percentage of adherent cells = (CFU/mL of recovered adherent bacteria ÷ CFU/mL of inoculum) × 100, and bars represent the fold-change relative to percent adhesion of control, with error bars representing ± one standard deviation. The Student *t-*tests were used to test for significance in comparison to the control (Bifidobacteria). *, *p*-value < 0.05; n.s., not significant.

Bacteria were incubated with IGEP (5 mg/mL) which was suspended in McCoy’s 5A tissue culture media as per Quinn et al. [18]. IGEP-treated *B. infantis* displayed a 3-fold increase in adhesion to HT-29 cells (Figure 2). Bovine serum-derived IgG (S-IgG) had no significant effect on *B. infantis* adhesion. Treatment of IGEP with sodium metaperiodate (MP-IGEP) to disable biological recognition of the conjugated oligosaccharides in the sample abolished any increase in *B. infantis* adhesion (*p*-value = 0.3773) [21]. Thus, the increase of adhesion of IGEP-treated *B. infantis* to HT-29 cells was mediated at least in part by IGEP glycosylation.

### 2.3. Combined Effect of IGEP and B. infantis on C. jejuni Adhesion

**Figure 3:**
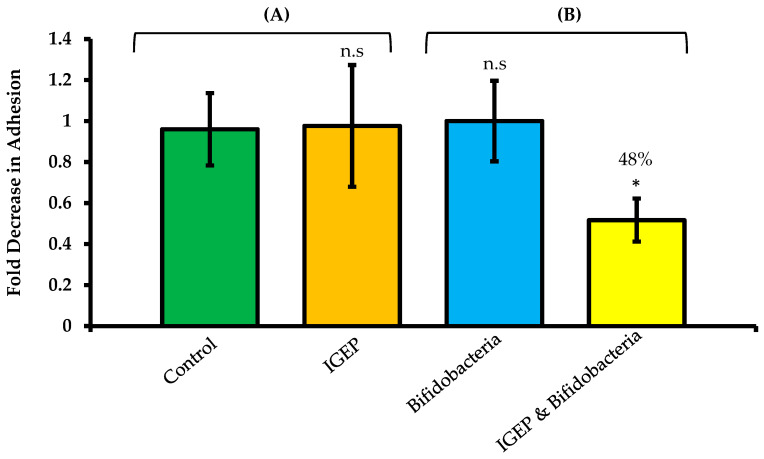
Anti-infective assays (**A**) demonstrating *Campylobacter jejuni* 81–176 adhesion in the absence (green) and presence (orange) of IGEP. (**B**) Competitive exclusion assays demonstrating *C. jejuni* 81–176 adhesion to HT-29 cells following pre-treatment of the HT-29 cells with *B. infantis* 15697 (blue) and *B. infantis* 15697 pre-treated with IGEP (yellow). Results are represented as the average of one biological replicate experiment performed in technical triplicate (**A**), and the average of three biological replicate experiments performed in technical triplicate (**B**), and are represented as a percentage of adherent cells = (CFU/mL of recovered adherent bacteria ÷ CFU/mL of inoculum) × 100, and graphed as fold-change relative to percent adhesion of control, with error bars representing ± one standard deviation. The Student *t*-tests were used to test for significance in comparison to the control (**A**), or in comparison to un-treated bifidobacteria (**B**). *, *p*-value < 0.05; n.s., not significant.

Anti-infective and competitive exclusion assays were employed here to investigate if (A) IGEP had a direct anti-infective effect on *C. jejuni* colonization, and (B) to determine if the increase in *B. infantis* adhesion as a result of IGEP pre-treatment resulted in a protective effect against *C. jejuni* colonization. An average inoculum of 4.7 × 10^8^ colony-forming units (CFU)/mL of *C. jejuni* were applied to the HT-29 cells, of which 3.1 × 10^5^ CFU/mL (0.1% ± 0.01% of the original inoculum) attached to the HT-29 cells. We chose *Campylobacter jejuni* 81–176 (*C. jejuni*) for this study as our group has previously demonstrated that bovine milk oligosaccharides have anti-infective effects against this strain [22]; additionally, it has been used in many other previous studies and is well characterized [19,21]. A high inoculum of *C. jejuni* was used in order to increase the opportunity of pathogenic infection of the HT-29 cells. Anti-infective assays were implemented as previously described [22] to determine if IGEP had any direct anti-pathogenic effects. No direct anti-infective effect was observed (*p*-value > 0.5) (Figure 3A). Competitive exclusion assays were implemented in order to assess if the increased adhesion observed in IGEP-treated *B. infantis* facilitated protection against pathogen colonization. First, the *B. infantis* was treated with IGEP for one hour, after which the bacteria were washed and applied to the HT-29 cells for 2 h. The cell line was washed to remove non-adherent *B. infantis* and then challenged with *C. jejuni* for 3 h prior to enumeration of colonized *C. jejuni*. Notably, IGEP-treated *B. infantis* revealed a statistically significant reduction in *C. jejuni* colonization following prior treatment of the HT-29 cells with IGEP-treated *B. infantis* (Figure 3B), with an average significant decrease of 48% in *C. jejuni* adherence observed. Neither *B. infantis* nor IGEP alone had protective effects against *C. jejuni* colonization (Figure 3A,B). Notably, no growth of *C. jejuni* in the presence of IGEP was observed under anti-infective and exclusion assay conditions. Overall, IGEP-treated *B. infantis* appears to provide prophylactic protection against *C. jejuni* colonization (48% reduction); this is likely through the increased colonization potential of *B. infantis*. Therefore, the combined use of IGEP and *B. infantis* may have superior bioactive potential compared to either used alone.

### 2.4. B. infantis Growth in IGEP

**Figure 4:**
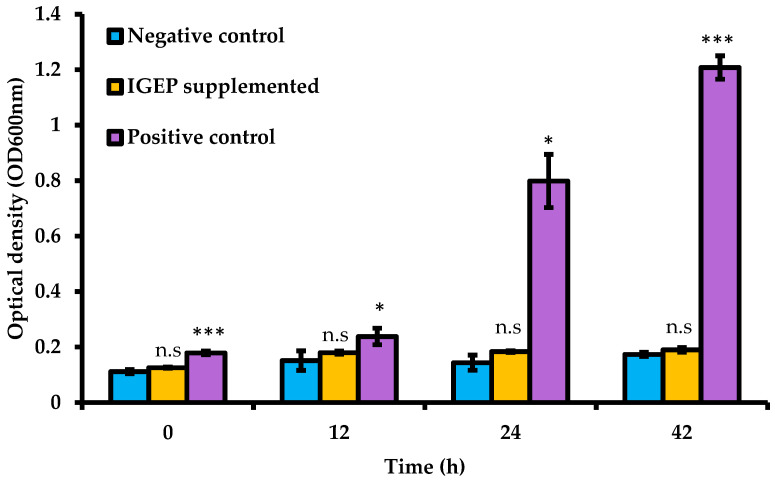
Growth of *B. infantis* in carbohydrate-free media supplemented with IGEP over 48 h (IGEP-supplemented) compared to a positive control of culture in de Man–Rogosa–Sharpe (MRS) with glucose and a negative control of non-supplemented carbohydrate-free media. Optical density at 600 nm was measured at 0, 6, 12, 24, and 48 h post-inoculation. Results are represented as the average of three replicates, with error bars representing ± one standard deviation. The Student *t*-tests were used to test for significance in comparison to the negative control. *, *p*-value < 0.05; ***, *p*-value < 0.002; n.s., not significant.

In order to investigate if growth was implicated in the observed increase in adhesion and consequent anti-infective activities, the ability of IGEP to stimulate the growth of *B. infantis* was examined. When IGEP was incubated with *B. infantis* over 42 h, no significant increase in bacterial growth was observed (Figure 4). Thus, it is unlikely that increased numbers of *B. infantis* contributed to the observed increase in adhesion to the HT-29 cells after exposure to IGEP.

### 2.5. B. infantis Metabolite Analysis

The presence of bifidobacteria in the gut influences the production of bacterial metabolites including formate, acetate, ethanol, and lactate [23,24]. In this study, HPLC analysis was used to quantify these metabolites in the supernatant following *B. infantis* growth in the presence of IGEP (Table 1). There were no statistically significant changes in levels of acetate, lactate, formate, or ethanol in IGEP-treated culture compared to no IGEP supplementation (control) following a 24 h fermentation, and the ratios of acetate to lactate (4:1) and acetate to formate (2:1) were the same for both the supplemented and non-supplemented samples. Ratios observed were likely due to carbohydrate (glucose) in the baseline media (McCoy’s media), as these assays were performed under adhesion assay conditions. Slight ethanol production was observed in the IGEP-supplemented sample (1:1), while no ethanol was detected in the non-supplemented sample, and this can likely be attributed to the IGEP composition itself. 

## 3. Discussion

In this study, IGEP increased the adhesion of *B. infantis* 3-fold to HT-29 cells. Notably, SDS-PAGE revealed that IgG was a dominate protein in the powder, although other Igs such as IgA and IgM may also have been present. IgG is present in human (0.1 g/L) and bovine (1.8 g/L) milk [25,26,27] and immunoglobulins (Igs) represent about 10–15% of whey proteins in both milk types [28,29]. Igs are known for their potent immunological properties and ability to inhibit gastrointestinal pathogens such as bacteria, protozoa, and viruses [30,31]. IgG is the predominant Ig in bovine milk and is known to be heavily glycosylated [32] and, in this respect, may also be expected to alter colonization of specific types of bacteria. Indeed, IgG is also the most common Ig in human blood serum [33]. IgG plays a crucial role in protective immunity and is involved in opsonization, agglutination, antibody-dependent cell-mediated cytotoxicity, and complement-dependent cytotoxicity activation [34,35]. Three domain structures exist in IgG; two structures are involved in antigen binding (Fab region) and the other is the fragment crystallizable (Fc) region that activates Fcγ receptors (FcγRs) of leukocytes and the C1 component of complement. One glycosylation site at asparagine 297 (*N*-linked) within the Fc region of IgG exists, in contrast to the vast array of other immunoglobulin isotypes which have more than one glycosylation site within the Fc region [36]. The hypervariable region of the antigen-binding fragment (Fab) of IgG has also been shown to contain *N*-glycans [37]. Notably, SDS-PAGE analysis of IGEP displayed trace bands between the 116 and 55 kDa markers that could be IgA components. Increased levels of secretory IgA (SIgA) in stools have been associated with infant formula supplemented with probiotics [38]. The commensal *Bacteroides fragilis* has been previously demonstrated to modulate binding of IgA to the intestinal mucosa suggesting that IgA may facilitate host-microbial symbiosis [39]. Additionally, SIgA has been shown to bind to mucin-producing HT29-MTX (HT-29 cells treated with methotrexate). This binding ability was significantly less in non-mucin-producing HT-29 cells [40]. In the current study we did not use mucin-producing HT-29 cells. Moreover, while trace levels of IgA may be present, IgG appeared to be the dominant Ig. Interestingly, the concentration of IgA is lower in bovine milk (0.08 g/L) than in bovine blood serum (0.37 g/L) [41]. Thus, while further characterization of the IGEP is required to fully identify the active component, considering the concentrations of IgA present in bovine milk and the results obtained via SDS-PAGE, it is more likely that IgG may be modulating the observed effects; however, isotyping is required to confirm this. Nonetheless, as periodate treatment abolished the increased adhesion of *B. infantis*, the glycan component of IGEP is likely to be responsible at least in part for the strain’s enhanced adhesion to the HT-29 cells.

The importance of milk protein glycosylation on biological function has been shown previously using glycomacropeptide (GMP). GMP, which promotes the growth of *B. infantis*, lost its prebiotic effect following periodate treatment [42]. Additionally, GMP’s ability to prevent *Escherichia coli* and *Salmonella enteritidis* intestinal infection was reduced following sialidase treatment and abolished following periodate oxidation [43]. Notably, bovine IgG-glycans contain fucose, galactose, and mannose structures in addition to sialic acids such as *N*-acetylneuraminic acid (Neu5Ac) and the non-human *N*-glycolylneuraminic acid (Neu5Gc) [44]. While IGEP-bound glycans may directly influence the adhesion of *B. infants*, it is important to note that *B. infantis* encodes an endoglycosidase, EndoBI-1 (glycosyl hydrolase family 18), which is capable of cleaving *N*-linked oligosaccharides on glycoproteins. This activity could potentially release oligosaccharides bound to glycoproteins in IGEP [45], resulting in the production of intact free oligosaccharides that could then directly influence the adhesion potential of the strain. Notably, in this study, bovine serum-derived IgG failed to induce any increase in the adhesion of *B. infantis*, suggesting that bovine milk-derived IgG may possess different glycan structures. Glycosylation is known to be cell-type specific [46,47] and IgG produced in different cells may contain differences in their oligosaccharide chains. In addition, the glycosylation pattern of milk-derived IgG is known to change over lactation [44] and may in part explain the different activities observed between serum- and milk-derived IgG.

IGEP may be influencing adhesion in a number of ways. For instance, it may induce the increased expression of adhesion factors by the bacteria. Our group previously demonstrated that milk oligosaccharides resulted in the increased adhesion of *B. infantis* to HT-29 cells. Exposure to the milk oligosaccharides was associated with an upregulation of genes involved in adhesion, e.g., DNA-binding protein-ferritin, GroEL, DnaK, and TadE, and a downregulation of genes involved in complex oligosaccharide metabolism [17]. IGEP may also aid the adhesion of *B. infantis* to the host cells by acting as a bridge between the bacteria and the host cells. For example, bovine lactoferrin has been shown to function as a molecular bridge for internalization of *Streptococcus uberis* into bovine mammary epithelial cells [48]. Less likely is the possibility that residual IGEP is modulating the cell line itself. This is unlikely as IGEP was washed from bacterial cells prior to exposure to intestinal cells. Our group has, however, recently shown that IgG isolated from bovine colostrum and milk does modulate the cell surface of HT-29 cells, in turn enhancing the adhesion of bifidobacteria to HT-29 cells [49].

A variety of milk-derived proteins have been shown to possess anti-infective activities. For example, lactoferrin has antimicrobial activity [50] and displays anti-adhesive effects against pathogens such as *E. coli* and enteropathogenic Y*ersinia* [51,52]. Lactoferrin also has been reported to promote the growth of various lactobacilli [53]. Previously, milk-fat globule membrane glycoproteins have been shown to have anti-adhesive properties against a range of pathogens, including rotavirus and various enteric bacteria [54,55]. Notably, sialic acid on some milk glycoproteins have been implicated in binding *E. coli* and *Salmonella enteritidis* [43,56], increasing growth of the bifidobacterial spp. such as *B. breve*, *B. bifidum*, B infantis [57], and preventing *Helicobacter pylori* colonization [58]. IgG in milk is responsible for agglutinating bacteria, neutralizing toxins, deactivating viruses, binding pathogens such as *Shigella flexneri*, *E. coli*, *Clostridium difficile,* and rotavirus [59,60], and developing an environment favorable for the growth of health-promoting bacteria [61]. In this study, 0.1% ± 0.01 of *C. jejuni* of the original inoculum adhered to the HT-29 cells, which is similar to the 0.25% ± 0.05 observed in a previous study by our group [19]. Interestingly, no protective effect was observed for IGEP alone, which was unexpected as IgA in breast milk has been associated with lower rates of *Campylobacter* in children [62]. This may be a result of the short exposure period of the pathogen to the IGEP throughout the anti-infective assays.

*C. jejuni* infection is prevalent in commercial broiler chickens in addition to swine, cattle, and contaminated water, and these sources act as a major reservoir that can result in human infection [63]. In this study, a combination of *B. infantis* and whey protein-derived IGEP resulted in a 48% decrease in pathogen colonization following prior treatment of the HT-29 cells with IGEP-treated *B. infantis.* Notably, the non-supplemented *B. infantis* control demonstrated no protective effect against *C. jejuni* colonization, in line with previous results that suggested a critical number of attached bifidobacteria are required to competitively exclude *C. jejuni* [19]. IGEP-treated *B. infantis* may result in the appropriate numbers interacting with the cell, which, in turn, may result in modulation of the HT-29 cells. *C. jejuni* is known to enter the gut epithelial cells and impair intestinal barrier function through cleavage of occludin by serine protease HtrA [64]. Notably, HMO-grown *B. infantis* is associated with a higher adhesion rate to HT-29 cells and has been shown to influence the expression of the epithelial cell surface receptors and immune responses [16]. In addition, occludin expression in Caco-2 cells is higher when exposed to HMO-grown *B. bifidum* [16]. Thus, protection against *C. jejuni* colonization could result from modulation of gene expression on the epithelial cells through exposure to IGEP-treated *B. infantis*. Previously, we demonstrated that an increase in adhesion induced by GMO had a protective effect against *C. jejuni* colonization of HT-29 cells in pathogen exclusion assays [19]. While no direct anti-infective effect was observed in this study, the concept that probiotics and pathogenic strains compete in the gastrointestinal tract for host cells surface receptors and nutrients has been well documented [65,66]. Bacteria such as *Lactobacillus* spp., i.e., *acidophilus*, *casei*, *crispatus*, *gasseri*, *helveticus*, *pentosus*, *plantarum*, *rhamnosus*, and *salivarius* have been suggested to exhibit anti-*Campylobacter* activities in vitro and in vivo [63,67]. In vivo studies using galactooligosaccharides/*B. longum* PCB 133 synbiotics have shown potential in reducing *C. jejuni* infection in poultry [68]. In addition, a microencapsulated *B. longum* PCB133 and xylooligosaccharides synbiotic can protect against *C. jejuni* infection in broiler chickens [69], and *L. salivarius* subsp. *salicinius* combined with 0.04% mannan oligosaccharides have demonstrated a decrease (3 log) in broiler chicken cecal *Campylobacter* levels [70]. While previous studies focused on pathogen protection using free glycans in synbiotics combinations, the use of milk glycoproteins also holds promise.

This study is particularly relevant considering the prevalence of *Campylobacter* and the fact that it is recognized as one of four key global causes of diarrheal diseases. Indeed, it is considered to be the most common bacterial cause of human gastroenteritis in the world and has an associated risk of secondary post-infection diseases such as irritable bowel syndrome and Guillain-Barre syndrome [21,71,72]. Further studies with other probiotic strains and pathogens are required to determine if this bioactivity can be extended to protect against other diseases.

IGEP did not demonstrate any significant influence on the growth of *B. infantis* or metabolite production following 24 h incubation with the strain and no statistically significant difference between the supplemented sample and non-supplemented sample were observed. This was somewhat unexpected as IGEP contains glycoproteins, and *B. infantis* has evolved to utilize milk glycans [73]. However, metabolite analysis showed growth on IGEP resulted in slightly higher levels of acetate, lactate and decreased levels of formate (Table 1). Additionally, low levels of ethanol, which were not detected in the non-supplemented sample were also observed. This may suggest that the level of glycans present in IGEP are too low to support growth. Notably, previous work in our lab has shown that growth on oligosaccharides isolated from goats milk results in slight ethanol production by bifidobacteria [19]. Therefore, this result is not surprising as the presence of bifidobacteria in the gut has been demonstrated to influence the production of formate, acetate, ethanol and lactate [23], and some bifidobacteria convert pyruvic acid into formic acid and ethanol as opposed to lactic acid, yielding an additional ATP [24]. Overall, these data indicate that IGEP did not significantly influence *B. infantis* metabolite production in vitro and that the altered adhesion of *B. infantis* when cultured in the presence of IGEP was not due to growth of the strain.

## 4. Materials and Methods

### 4.1. Materials and Bacterial Strains

The IGEP was kindly provided by Upfront Chromatography (Copenhagen, Denmark). The protein content of the powder was determined by the Bradford assay [20]. *B. longum* subsp. *infantis* ATCC^®^ 15697™ (*B. infantis*) was obtained from the American Type Culture Collection (ATCC, Middlesex, UK). *C. jejuni* 81–176 was kindly provided by Dr Marguerite Clyne’s, University College Dublin.

### 4.2. Sodium Dodecyl Sulfate-Polyacrylamide Gel Electrophoresis (SDS-PAGE) Analysis

Sample preparation and reduction was performed as per the manufacturer’s instructions (NuPAGE system, Life Technologies, Thermo Fisher Scientific Inc.). In brief, 7.5 μL of sample buffer, 3 μL of reducing agent, and 12 μL of deionized water were added to 7.5 μL of sample at 10 mg/mL (by mass of powder) to give a final volume of 30 μL and a total of 15 μg of protein/well. The sample was then centrifuged and heated to 70 °C for 10 min, and 10 μL of sample was added to each well of a 4–12% Bis-Tris gel (1.00 mm × 9 well, Life Technologies). A molecular weight standard solution (Invitrogen Mark12 Unstained Standard, Thermo Fisher Scientific Inc.), was prepared as per the manufacturer’s instructions (diluted 1:10 with Invitrogen LDS Sample Buffer) and loaded onto the gel. Electrophoresis was performed at 200 V for 50 min using MOPS buffer supplemented with 0.25% NuPAGE Antioxidant (Life Technologies) in the upper chamber. Protein bands were visualized on the gels using Coomassie Blue stain (Invitrogen SimplyBlue SafeStain) following the manufacturer’s procedure.

### 4.3. Periodate Treatment of Powder

Sodium metaperiodate (NaIO_4_) treatment of IGEP was performed to oxidize IGEP andproduce IGEP-P as previously described [21]. In brief, IGEP (10 mg/mL) was incubated with 0.011 mM NaIO_4_ at 4 °C for 30 min. To remove excess NaIO_4_, the sample was centrifuged using a 3 kDa MWCO (Amicon) with three washes of phosphate-buffered saline at pH 7.4 (PBS). The retentate collected contained the IGEP and was lyophilized to produce a powder. A negative control containing no IGEP, and a control sample not treated with NaIO_4_ were also included.

### 4.4. Bacterial Culture

Bacterial cultures were maintained as previously described [17,18]. *B. infantis* was stored in de Man–Rogosa–Sharpe (MRS) (Difco, Sparks, MD, USA) broth containing 50% glycerol at −80 °C. The strain was cultured twice in MRS media supplemented with L-cysteine (0.05% *w*/*v*) (Merck, Dannstadt, Germany) prior to use, and was routinely grown overnight at 37 °C under anaerobic conditions generated using an Anaerocult A system (Merck, Dannstadt, Germany).

*C. jejuni* 81–176 was stored in Mueller Hinton broth (Oxoid, Ireland c/o Fannin Healthcare, Dublin, Ireland) containing 50% glycerol at −80 °C and cultured directly from storage onto Mueller-Hinton agar plates. The pathogen was grown under microaerophilic conditions generated using CampyGen gas packs (Oxoid), for 48 h at 37 °C as previously described [19,22]. Prior to pathogen inhibition assays, *C. jejuni* 81–176 was grown on Mueller-Hinton agar and then transferred to biphasic media in 25 cm^2^ tissue culture flasks (Corning, New York, NY, USA) consisting of Mueller-Hinton agar supplemented with *Campylobacter* selective supplement (Skirrow), (Oxoid) and 6 mL of McCoy’s 5A media (Merck) supplemented with 2% fetal bovine serum (FBS). The flask was then incubated for 24 h under microaerophilic conditions at 37 °C.

### 4.5. Exposure of B. infantis to IGEP for Adhesion Assays

Exposure of the bacteria to IGEP was performed as previously described [18,19]. Bacteria were used at mid-exponential growth phase (18 h), and the OD_600nm_ was adjusted to 0.3 at the start of the assay, after which the cells were cultured for 1.5–2 h and used once an OD_600nm_ of 0.5 was reached (corresponding to approximately 1.6 × 10^8^ CFU/mL). Bacterial cells were washed twice with PBS by centrifugation (3850× *g*, 5 min). Cell pellets were re-suspended to a final OD_600nm_ of 0.25 in non-supplemented, or IGEP-supplemented McCoy’s 5A tissue culture media. A final concentration of 5 mg/mL as per Quinn et al. [18] was used. Bacterial suspensions were then incubated for 1 h at 37 °C under anaerobic conditions. Following this, bacteria were harvested by centrifugation (3850× *g*, 5 min), the supernatants removed, and pellets were washed three times in PBS and then re-suspended in non-supplemented McCoy’s media prior to use in the adhesion assays.

### 4.6. Mammalian Cell Culture Conditions

HT-29 cells were grown as previously described [18,19] and maintained in McCoy′s 5A modified medium (Merck) supplemented with 10% FBS using 75 cm^2^ tissue culture flasks incubated at 37 °C in 5% CO_2_ in a humidified atmosphere. Once the cells were nearing confluency (approximately 80–90%), they were passaged into 48-well tissue culture plates (Sarstedt Ltd., Wexford, Ireland) at a density of 1 × 10^5^ cells/mL between passages 15–21. The cells were then used once fully confluent (approximately 2 × 10^6^ cells/well). The media was changed every other day and supplemented with 2% FBS 24 h prior to use.

### 4.7. Adhesion Assays with B. infantis

Adhesion assays with *B. infantis* were performed as previously described [18,19]. HT-29 cells were washed twice with PBS, and 250 µL of the bacteria and media suspensions were added to the wells, corresponding to approximately 40 bacterial cells per human cell. Bacterial cells were incubated with the HT-29 cells for 2 h at 37 °C under anaerobic conditions using an Anaerocult A system (Merck). The HT-29 cells were then washed five times with PBS to remove non-adherent bacteria. HT-29 cells were then lysed with 250 µL of 1% TritonTM X-100 (Merck) for 5 min at 37 °C. The lysates were serially diluted and enumerated by spot-plating on MRS plates to enumerate bacterial colony-forming units (CFU). The adhesion of the bacteria was determined as the percentage of original inoculum which attached, thus accounting for variations in the starting inoculum. Percentage adhesion = (CFU/mL of recovered adherent bacteria/CFU/mL of inoculum) × 100. Experiments were performed in triplicate on three separate occasions.

### 4.8. Anti-Infective Assays and Exclusion Assay

Anti-infective assays were performed as previously described [19,22]. In brief, *C. jejuni* was incubated in the absence and presence of IGEP (5 mg/mL) at a final OD_600nm_ of 0.3 (approximately 4.7 × 10^8^ CFU/mL) in McCoys’s media and incubated under microaerophilic conditions for 1 h at 37 °C; 250 µL of the mix was then applied to three wells containing HT-29 cells, and allowed to incubate for 3 h, after which the eukaryotic cells were washed five times with PBS, lysed with 250 µL 0.1% Triton X-100 in PB, plated onto Mueller-Hinton agar plates, and incubated under microaerophilic conditions for 72 h at 37 °C to enumerate CFU. For exclusion assays, exposure of *B. infantis* to 5 mg/mL IGEP was performed as described above, and this suspension was subsequently incubated with the HT-29 cells for 2 h. A non-supplemented control was also included. Non-adherent bacteria were removed from the cells as described above, after which the cell line was challenged with *C. jejuni*. To do this, the pathogen was harvested from the biphasic medium, washed twice in non-supplemented McCoy’s, and diluted to an OD_600nm_ of 0.3. From this suspension, 250 μL was then added to each well, and cells were incubated under anaerobic conditions for 3 h at 37 °C. Cells were then washed five times with PBS, lysed with 0.1% Triton X-100 in PBS, and plated onto supplemented Mueller-Hinton agar. Mueller-Hinton plates were incubated under microaerophilic conditions for 72 h at 37 °C, after which bacterial CFU were enumerated. For anti-infective assays and competitive exclusion assays, all results are presented as the mean of biological replicate experiments, with error bars representing standard deviation, graphed as fold-change relative to percent adhesion of the control. Percentage adhesion = (CFU/mL of recovered adherent bacteria ÷ CFU/mL of inoculum) × 100.

### 4.9. Effect of IGEP on the Growth of B. infantis

*B*. *infantis* was grown in the absence and presence of 5 mg/mL IGEP over a 48 h period under adhesion assay conditions and in carbohydrate-free growth medium; de Man–Rogosa–Sharpe (MRS) supplemented with L-cysteine (0.05% *w*/*v*) was prepared by the first principles excluding glucose and meat extract [74,75]. Carbohydrate-free MRS was unable to support bacterial growth above an OD_600nm_ of 0.2. The optical density readings between supplemented and un-supplemented carbohydrate-free media were noted and the difference between these was recorded as the turbidity of the sample, and this was added to all control sample readings to correct the difference in optical density. Aliquots of 150 μL of the bacterial suspensions were added to the individual wells of a 96-well microtiter plate corresponding to an optical density of 0.03. Other controls included a control containing no bacteria, and bacteria were grown in MRSbroth supplemented with L-cysteine (0.05% *w*/*v*) (Merck). These experiments were performed in an anaerobic hood and bacterial growth was monitored by determining absorbance (OD_600nm_) at 0, 12, 24, and 48 h. The microtitre plate was automatically shaken for 30 s prior to each measurement to achieve a homogenous suspension. Experiments under adhesion assay conditions were performed in triplicate on three separate occasions and in triplicate under carbohydrate free-MRS conditions. The percentage change in growth was calculated as the difference in OD_600nm_ between the supplemented and non-supplemented samples.

### 4.10. B. infantis Metabolite Analysis

Metabolite analysis was conducted as previously described [19]. *B. infantis* was grown overnight under the optimal conditions outlined above and then re-suspended in McCoy’s media at an OD_600nm_ of 0.25 with a final oligosaccharide concentration of 5 mg/mL. A one milli-litre aliquot of this cell suspension was then dispensed into a sterile Eppendorf after 0 and 24 h of growth. A negative control containing no bacteria was also included. These solutions were then centrifuged for 5 min (3850× *g*) and the supernatants collected. The process was repeated three times to ensure that bacteria were not present in the supernatant. The sample was also treated with ultraviolet light for 30 min in a laminar flow hood to ensure no further metabolic activity occurred [19]. The metabolic-end products (lactate, acetate, formate, and ethanol) in the supernatant were measured in triplicate using an Agilent 1200 HPLC system (Agilent Technologies, Santa Clara, CA, USA) with a refractive index detector. A negative control of non-supplemented media was also included. A REZEX 8 m 8% H, organic acid column (300 × 7.8 mM Phenomenex, CA, USA) was used and the elution was performed for 25 min with a 0.01 M H_2_SO_4_ solution at a constant flow rate of 0.6 mL/min and a temperature of 65 °C. Metabolite peaks and concentrations were identified and calculated based on known metabolite retention times and standard solutions at known concentrations. The ratios of acetate to lactate, acetate to formate, and lactate to ethanol were also calculated.

### 4.11. Statistical Analysis

Graphs were drawn using Microsoft Excel. The results are presented as the mean ± standard deviations of replicate experiments, and the Student *t*-tests were used to determine statistically significant results in comparison to the control. For all experiments, *p*-value < 0.05 was considered significant.

## 5. Conclusions

Exposure of *B. infantis* to IGEP resulted in an increased attachment to HT-29 cells and was in part mediated by IGEP glycosylation. This synbiotic combination ultimately led to protection against *C. jejuni* colonization of HT-29 cells in vitro. This is a particularly beneficial outcome as *Campylobacter* is recognized in both developed and developing counties as a leading cause of diarrheal diseases and is most common in children younger than five years of age [71,76]. Animal reservoirs are the most common source of *Campylobacter* infection, with 60% to 80% of broiler chicken flocks estimated to be contaminated with *Campylobacter* at slaughter [77], and a suggested 48% of *Campylobacter* infections are thought to be related poultry exposure [78]. Consequently, host protection via competitive exclusion by commensals has the potential to reduce the rising numbers of *Campylobacter* infections in humans and could be extended for use in livestock animal feed to reduce the rates of transmission from such reservoirs. Moreover, the use of synbiotics such as IGEP in combination with probiotic bacteria have potential to be used as a means to protect against other gastrointestinal pathogens. Overall, this study highlights the benefits of using a combination of probiotic bacteria and IgG-enriched whey to promote overall gut health, and indicates that synbiotic combinations may hold superior health-promoting properties when compared with either probiotics or prebiotics when used alone.

## Figures and Tables

**Table 1:** Metabolite analysis by *B. infantis* following 24 h incubation with IGEP. Concentration values (mM) are the average of three biological replicates, each analyzed in technical triplicate.

Concentration (mM)	Control	IGEP
Acetate	2.42	2.70
Lactate	0.62	0.77
Formate	0.32	0.26
Ethanol	ND	1.15

ND; not detected.
